# Nickel nanoparticle-induced cell transformation: involvement of DNA damage and DNA repair defect through HIF-1α/miR-210/Rad52 pathway

**DOI:** 10.1186/s12951-021-01117-7

**Published:** 2021-11-17

**Authors:** Yiqun Mo, Yue Zhang, Yuanbao Zhang, Jiali Yuan, Luke Mo, Qunwei Zhang

**Affiliations:** Department of Environmental and Occupational Health Sciences, School of Public Health and Information Sciences, University of Louisville, 485 E. Gray Street, Louisville, KY 40202 USA

**Keywords:** Nickel nanoparticles (Nano-Ni), DNA damage, HIF-1α, miR-210, Rad52, Cell transformation

## Abstract

**Background:**

Nickel nanoparticles (Nano-Ni) are increasingly used in industry and biomedicine with the development of nanotechnology. However, the genotoxic and carcinogenic effects of Nano-Ni and the underlying mechanisms are still unclear.

**Methods:**

At first, dose–response (0, 10, 20, and 30 μg/mL) and time-response (0, 3, 6, 12, and 24 h) studies were performed in immortalized normal human bronchial epithelial cells BEAS-2B to observe the effects of Nano-Ni on DNA damage response (DDR)-associated proteins and the HIF-1α/miR-210/Rad52 pathway by real-time PCR or Western blot. Then, a Hsp90 inhibitor (1 µM of 17-AAG, an indirect HIF-1α inhibitor), HIF-1α knock-out (KO) cells, and a miR-210 inhibitor (20 nM) were used to determine whether Nano-Ni-induced Rad52 down-regulation was through HIF-1α nuclear accumulation and miR-210 up-regulation. In the long-term experiments, cells were treated with 0.25 and 0.5 µg/mL of Nano-Ni for 21 cycles (~ 150 days), and the level of anchorage-independent growth was determined by plating the cells in soft agar. Transduction of lentiviral particles containing human Rad52 ORF into BEAS-2B cells was used to observe the role of Rad52 in Nano-Ni-induced cell transformation. Nano-Ni-induced DNA damage and dysregulation of HIF-1α/miR-210/Rad52 pathway were also investigated in vivo by intratracheal instillation of 50 µg per mouse of Nano-Ni. *gpt* delta transgenic mice were used to analyze mutant frequency and mutation spectrum in mouse lungs after Nano-Ni exposure.

**Results:**

Nano-Ni exposure caused DNA damage at both in vitro and in vivo settings, which was reflected by increased phosphorylation of DDR-associated proteins such as ATM at Ser1981, p53 at Ser15, and H2AX. Nano-Ni exposure also induced HIF-1α nuclear accumulation, miR-210 up-regulation, and down-regulation of homologous recombination repair (HRR) gene Rad52. Inhibition of or knocking-out HIF-1α or miR-210 ameliorated Nano-Ni-induced Rad52 down-regulation. Long-term low-dose Nano-Ni exposure led to cell malignant transformation, and augmentation of Rad52 expression significantly reduced Nano-Ni-induced cell transformation. In addition, increased immunostaining of cell proliferation markers, Ki-67 and PCNA, was observed in bronchiolar epithelial cells and hyperplastic pneumocytes in mouse lungs at day 7 and day 42 after Nano-Ni exposure. Finally, using *gpt* delta transgenic mice revealed that Nano-Ni exposure did not cause increased *gpt* mutant frequency and certain DNA mutations, such as base substitution and small base insertions/deletions, are not the main types of Nano-Ni-induced DNA damage.

**Conclusions:**

This study unraveled the mechanisms underlying Nano-Ni-induced cell malignant transformation; the combined effects of Nano-Ni-induced DNA damage and DNA repair defects through HIF-1α/miR-210/Rad52 pathway likely contribute to Nano-Ni-induced genomic instability and ultimately cell transformation. Our findings will provide information to further elucidate the molecular mechanisms of Nano-Ni-induced genotoxicity and carcinogenicity.

**Graphical Abstract:**

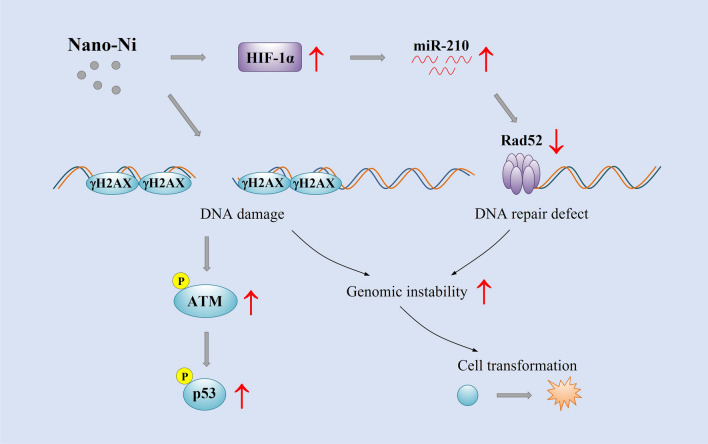

**Supplementary Information:**

The online version contains supplementary material available at 10.1186/s12951-021-01117-7.

## Background

As nanotechnology advances, an increasing number of metal nanoparticles are being developed, manufactured, and applied. Nickel nanoparticles (Nano-Ni) are an important class of transition metal nanoparticles that have increasing use in a range of industrial and biomedical fields, including drug and gene delivery, magnetic resonance imaging (MRI), biomedical detection, diagnostics electronics, as a catalyst, adsorption of dyes, solar cells and sensors, superconductors, etc. [1]. The expanding production and use of Nano-Ni poses an increased risk of human health effects and environmental contamination at both occupational and non-occupational settings. There are growing concerns over their adverse effects at their portals of entry, including the lungs, the skin, and the gastrointestinal tract. We and other groups have demonstrated that exposure to Nano-Ni causes mouse or rat lung inflammation, injury, and fibrosis [2–7], and also induces matrix metalloproteinases MMP-2 and MMP-9 production in mouse lungs and peripheral blood monocytes and human monocytes U937 [2, 3, 8, 9]. Exposure of human epidermal keratinocytes HaCaT to Nano-Ni caused dysregulation of tight junction-associated proteins [10]. There are reports of Nano-Ni exposure causing adverse health effects in humans. For example, a chemist developed nickel sensitization while weighing Nano-Ni powder without any special protective or control measures [11]. Accidental exposure to Nano-Ni caused a previously healthy individual to develop adult respiratory distress syndrome (ARDS) and death after inhaling ~ 1 g of Nano-Ni from occupational exposure [12]. Furthermore, nickel and nickel compounds have carcinogenic effects [13]. According to the International Agency for Research on Cancer (IARC) monographs, nickel compounds are listed as Group 1 carcinogens (carcinogenic to humans), while metallic nickel and nickel alloys are Group 2B (possibly carcinogenic to humans) [14]. However, the potential genotoxic and carcinogenic effects of Nano-Ni are still unclear. In this study, we explored whether exposure to Nano-Ni could induce DNA damage, DNA repair defects, and cell transformation and the possible mechanisms involved in these processes.

Exogenous and endogenous stressors may damage nuclear DNA, which include mismatches due to replication, single-strand DNA breaks (SSBs), double-strand DNA breaks (DSBs), etc. These lesions contribute to cellular malfunction and the onset of diseases including cancer [15, 16]. Among them, DSBs are more lethal to cells and require rapid countermeasures to ensure cell survival. Different types of DNA damage trigger specific DNA damage response (DDR) pathways that detect, signal, and repair damaged DNA [15, 16]. The protein kinase ataxia-telangiectasia mutated (ATM) is a central signal transducer of DNA damage and is the primary responder to DNA DSBs, which is auto-phosphorylated at Ser1981 upon detection of DNA damage [17, 18]. Activated ATM modifies directly or indirectly a broad range of targets, including p53 and H2AX, resulting in their modification, such as phosphorylation, to propagate DDR signaling [19–21]. Our previous studies have found that exposure to cobalt nanoparticles (Nano-Co) caused DSBs, which was reflected by increased phosphorylation of ATM, p53, and H2AX [18]. However, whether Nano-Ni exposure could cause DNA damage, especially DSBs, is still unclear.

Hypoxia-inducible factor 1 (HIF-1) is a heterodimer consisting of a constitutively present β subunit and a short-lived, oxygen-regulated α subunit. Under normoxic conditions, prolyl hydroxylase domain enzymes (PHDs) hydroxylate proline residues on HIF-1α, allowing it to be recognized by Von Hippel-Lindau (VHL) protein, a part of an E3 ubiquitin ligase complex, and thereby targeted for degradation by the 26S proteasome. However, under hypoxic conditions, a decrease in PHD activity leads to HIF-1α accumulation, heterodimerization with the β-subunit, recruitment of the histone acetyltransferases p300 and CBP, and transactivation of target gene expression [22–24]. HIF-1 is a key integrator of cell signaling pathways that induce tumor angiogenesis, and an essential step for tumor growth [25–27]. Our previous studies have revealed that Nano-Ni exposure caused HIF-1α nuclear accumulation [9, 10]. It raises an intriguing question whether Nano-Ni-induced HIF-1α nuclear accumulation is involved in Nano-Ni-induced genotoxic and carcinogenic effects.

miRNAs are non-coding, single-stranded RNAs of ~ 22 nucleotides and constitute a novel class of gene regulators that are found in both plants and animals [28]. Previous studies have demonstrated that miRNAs are dynamically regulated in various human diseases, including pulmonary and cardiovascular diseases [29–34] and tumorigenesis [32, 35]. HypoxamiRs are a group of specific miRNAs, which are overexpressed during the hypoxic response in normal and transformed cells [36]. miR-210, a prototypical hypoxamiR, is overexpressed in most solid tumors and is linked to adverse prognosis in many tumor types [36]. Previous studies showed that miR-210 is a direct HIF-1 target and HIF-1 regulates the expression of miR-210 in a variety of tumor types through a hypoxia-responsive element (HRE) [36–40]. Since Nano-Ni exposure induces HIF-1α nuclear accumulation [9, 10], the effects of Nano-Ni on miR-210 expression and the relationship between miR-210 and HIF-1α are worth exploring.

Repair of DNA double strand breaks (DSBs) can be achieved by multiple pathways including homologous recombination (HR), non-homologous end joining (NHEJ), and other alternative repair pathways [41]. HR, the major error-free repair pathway, is extremely important for both cell survival and the maintenance of genomic integrity [41]. Rad52 is an important recombinational repair mediator. In HR, mammalian cells rely on BRCA2 to mediate loading of Rad51 onto replication protein A (RPA)-coated ssDNA, and on Rad52 for annealing ssDNA ends [42]. In a BRCA-deficient context, Rad51 loading can be carried out by Rad52, and simultaneous targeting of PARP1 and RAD52 triggers dual synthetic lethality in BRCA-deficient cells [43–45]. Overexpression of Rad52 in cultured monkey FSH2 cells significantly increases recombination frequencies following exposure to γ-rays [46]. A previous study showed that traffic fine particulate matter (PM_2.5_) exposure suppressed Rad52 expression in human lung A549 cells [47]. On the other hand, Rad52 is identified as a miR-210 target; overexpression of miR-210 suppresses the level of Rad52 [40, 48].

In this study, we investigated whether Nano-Ni exposure would cause DNA damage, DNA repair defects, and cell transformation, as well as the role of dysregulation of the HIF-1α/miR-210/Rad52 pathway in Nano-Ni-induced DNA damage and cell transformation at both in vitro and in vivo settings. Similar-sized titanium dioxide nanoparticles (Nano-TiO_2_) were used as a control since our previous studies showed that exposure to Nano-TiO_2_ did not induce either dysregulation of DNA damage-associated proteins [18, 49] or HIF-1α nuclear accumulation [9, 10].

## Materials and methods

### Nickel and titanium dioxide nanoparticles and their characterization

Nano-Ni (Lot No. 2237) and Nano-TiO_2_ (Lot No. TiO_2_-55–1) used in this study were obtained from Inabata & Co., Ltd., Vacuum Metallurgical Co., Ltd., Japan. Their characteristics were described previously [2, 7, 9, 50]. Briefly, Nano-Ni is composed of Ni (85–90%) and NiO (10–15%), while Nano-TiO_2_ is composed of anatase (90%) and rutile (10%) [2, 7, 50]. The mean diameters of Nano-Ni and Nano-TiO_2_ in the powder are 20 nm and 28 nm determined by transmission electron microscopy (TEM), and their mean hydrodynamic sizes are 250 nm and 280 nm determined by dynamic light scattering (DLS). The specific surface area is 43.8 m^2^/g for Nano-Ni and 45.0 m^2^/g for Nano-TiO_2_ [2, 7, 9]. Nano-Ni and Nano-TiO_2_ were dispersed in physiological saline, ultrasonicated for 10 min in an ultrasonic cleaner FS30 (Fisher Scientific, Pittsburg, PA), and vortexed thoroughly prior to each experiment.

### Chemicals and reagents

Anti-phospho-ATM (cat. no. 05–740) antibody was obtained from MilliporeSigma (Burlington, MA). Anti-ATM (cat. no. 2873), anti-phospho-p53 (cat. no. 9286 or 9284), and anti-β-actin (cat. no. 4970) antibodies were from Cell Signaling Technology (Beverly, MA). Anti-p53 (cat. no. sc-126) and anti-PCNA (cat. no. sc-7907) antibodies were from Santa Cruz Biotechnology (Santa Cruz, CA). Anti-p53 (cat. no. ab246550), anti-γH2AX (cat. no. ab26350 or ab81299), anti-Rad52 (cat. no. ab180721), and anti-HIF-1α (cat. no. ab463 or ab179483) antibodies were from abcam (Cambridge, MA). Anti-HIF-1α (cat. no. 610959) antibody was from BD (San Jose, CA). Anti-Ki-67 (cat. no. PAI-38032) antibody was from Thermo Fisher Scientific (Waltham, MA). HRP-conjugated goat anti-rabbit IgG (cat. no. 7074) or horse anti-mouse IgG (cat. no. 7076) were from Cell Signaling Technology (Beverly, MA). Biotinylated goat anti-rabbit IgG (cat. no. IR2145) was from ImmunoReagents (Raleigh, NC), while HRP-conjugated streptavidin (code: 016–030-0840) was from Jackson ImmunoResearch (West Grove, PA).

The mirVana™ miRNA inhibitor for has-miR-210-3p (assay ID: MH10516), mirVana™ miRNA inhibitor Negative Control #1 (cat. no. 4464076), and Lipofectamine™ RNAiMAX Transfection Reagent were purchased from Thermo Fisher Scientific (Waltham, MA). All reagents for cell culture including culture medium, fetal bovine serum (FBS), penicillin/streptomycin solution, and 0.05% Trypsin/0.53 mM EDTA were from Corning (Manassas, VA). All other chemicals were purchased from Fisher Scientific (Fair Lawn, NJ) unless otherwise indicated.

### Cell culture and nanoparticle treatment

Immortalized normal human bronchial epithelial cells BEAS-2B were obtained from American Type Culture Collection (ATCC, cat. no. CRL-9609, Manassas, VA) and cultured in RPMI 1640 medium supplemented with 10% FBS, 100 IU/mL penicillin, and 100 μg/mL streptomycin in an incubator with a humidified atmosphere of 5% CO_2_ at 37 °C. Hypoxia inducible factor 1α knock-out cells [HIF-1α (-/-)] and their wild-type cells [HIF-1α (+ / +)] were originally obtained from Dr. R. Johnson (University of California San Diego, San Diego, CA) and cultured in DMEM supplemented with 10% FBS, 100 IU/mL penicillin, and 100 µg/mL streptomycin.

For the short-term dose–response study, cells were treated with 5, 10, 20, and 30 μg/mL of Nano-Ni or Nano-TiO_2_ for 24 h. For the time-response study, cells were treated with 20 μg/mL of Nano-Ni for 3, 6, 12, and 24 h. To observe the effects of Nano-Ni-induced HIF-1α nuclear accumulation on miR-210 and Rad52 expression, BEAS-2B cells were pretreated with 1 µM of 17-(Allylamino)-17-demethoxygeldanamycin (17-AAG) (InvivoGen, San Diego, CA) for 4 h, followed by treatment with 20 µg/mL of Nano-Ni for 24 h. 17-AAG inhibits heat shock protein 90 (Hsp90), a HIF-1α chaperone, thus promoting HIF-1α degradation and diminishing HIF-1α transcriptional activity [51]. HIF-1α wild-type [HIF-1α (+ / +)] and knock-out [HIF-1α (-/-)] cells were treated with 20 µg/mL of Nano-Ni for 24 h.

For long-term Nano-Ni exposure, BEAS-2B cells were seeded in 75 cm^2^ flasks in 10 mL of complete medium. After culturing for 3–4 days, the medium was replaced with fresh complete medium containing 0.25 or 0.5 µg/mL of Nano-Ni. Cells without Nano-Ni treatment were used as control. After 3–4 days of Nano-Ni treatment, cells were split. This procedure was repeated 21 times (~ 150 days). During each split, cells were collected and stored at − 80 °C for later analyses such as Western blot, used for soft agar colony formation assay, or frozen in liquid nitrogen.

### Cytotoxicity of Nano-Ni and Nano-TiO_2_

The cytotoxicity of Nano-Ni and Nano-TiO_2_ was determined by two different methods. One method is CellTiter 96 AQ_ueous_ Non-Radioactive Cell Proliferation Assay (MTS assay) (Promega, Madison, WI), which is a colorimetric method for determining the number of metabolically active cells in which the dehydrogenase enzymes can convert a tetrazolium compound (MTS) into an aqueous, soluble, and colored formazan. Briefly, 5 × 10^3^ cells per well were seeded into 96-well plates and allowed to attach to the growth surface by culturing overnight. Then cells were treated with different concentrations (0, 5, 10, 20, 30, and 40 μg/mL) of Nano-Ni or Nano-TiO_2_ for 24 h. The cytotoxicity was determined according to the manufacturer’s instruction and our previous studies [9, 52]. Another method is the alamarBlue™ assay (Invitrogen, Eugene, OR), which is a colorimetric/fluorometric method to quantitatively measure the proliferation of cells by using the reducing power of living cells through an oxidation–reduction indicator. This method was performed according to the manufacturer’s instructions and our previous study [9].

### Protein extraction and Western blot

Nuclear protein was extracted from the cells using NE-PER® Nuclear and Cytoplasmic Extraction Reagent (Thermo Scientific, Rockford, IL) to detect the expression of DNA damage response-associated proteins, HIF-1α, and Rad52 in cells after Nano-Ni or Nano-TiO_2_ exposure according to the manufacturer’s instruction and our previous studies [10, 53]. Total protein from mouse lung tissues were isolated by using RIPA lysis buffer supplemented with PMSF, protease inhibitor cocktail, and sodium orthovanadate (Santa Cruz Biotechnology, Santa Cruz, CA) as described in our previous studies [3, 8]. Briefly, mouse lung tissues were homogenized on ice using a Tissue Tearor homogenizer (BioSpec Products, Bartlesville, OK), followed by using an ultrasonic cell disruptor (Fisher Scientific, Fair Lawn, NJ) to break down cell clusters. After setting on ice for 40 min and centrifuging at 12,000 g and 4 °C for 15 min, the supernatant was collected. The protein concentration was determined using Bio-Rad Protein Assay (Bradford method) (Bio-Rad, Hercules, CA) with a DU730 Spectrophotometer (Beckman Coulter, Fullerton, CA).

Western blot was performed as described in our previous studies [3, 54]. Immunoreactive bands were detected using SuperSignal™ West Pico PLUS Chemiluminescent Substrate (Thermo Scientific, Rockford, IL) followed by exposure to CL-XPosure™ film (Thermo Scientific). Equal nuclear protein loading was verified by Coomassie Brilliant Blue staining. For mouse lung proteins, the expression of β-actin was used as an internal reference. Immunoreactive bands were quantified using NIH ImageJ software (http://imagej.nih.gov/ij/).

### Total RNA isolation and real-time PCR

To determine the expression level of miR-210, total RNA was isolated from cultured cells or mouse lung tissues by using mirVana miRNA Isolation Kit (Abcam, Cambridge, MA) as described in our previous studies [3, 8]. The concentration of total RNA was measured by absorbance at 260 nm with a DU 730 Spectrophotometer (Beckman Coulter, Fullerton, CA). TaqMan® microRNA Assay for miR-210 was used (assay ID 000,512, Applied Biosystems, Foster City, CA). 10 ng total RNA per sample was reverse-transcribed using TaqMan® MicroRNA Reverse Transcription Kit (Applied Biosystems). Then, 2 µL RT product from each sample was used to perform real-time PCR using TaqMan® Universal PCR Master Mix (Applied Biosystems). Values of miR-210 expression were normalized to the expression of the endogenous control U6 snRNA (assay ID 001,973, Applied Biosystems) in the same sample and calculated using the 2^−ΔΔCT^ (Livak) method [55]. The results were reported as fold increase as compared to the control that was without metal nanoparticle exposure.

### Transduction of lentiviral particles containing human Rad52 ORF

To establish cells with stable overexpression of human Rad52 protein, BEAS-2B cells were infected with lentiviral particles containing human Rad52 ORF (cat. no. RC222194L3V, Origene, Rockville, MD) at a multiplicity of infection (MOI) of 10 according to the manufacturer’s instructions. 8 µg/mL of polybrene (MilliporeSigma, Burlington, MA) was used to enhance the transduction efficiency. After 20 h transduction, cells were trypsinized, 1:10 diluted, seeded into 10 cm dishes, and selected with 2 µg/mL of puromycin (VWR, Radnor, PA). 11 puromycin-resistant colonies were picked and expanded for Western blot analysis to confirm the overexpression of Rad52 protein. Cells of colony No. 1 was used for long-term Nano-Ni exposure and soft agar colony formation assay.

### Transfection of miR-210 inhibitor

To inhibit miR-210 expression, 2 × 10^5^ BEAS-2B cells were seeded in each well of 6-well plates in 2 mL antibiotic-free RPMI1640 supplemented with 10% FBS and cultured overnight. The mirVana™ miRNA inhibitor of has-miR-210-3p (20 nM) or Negative Control #1 (20 nM) was transfected into cells in antibiotic-free and FBS-free RPMI1640 by using Lipofectamine™ RNAiMAX Transfection Reagent according to the manufacturer’s protocol and our previous study [52]. After 24 h transfection, the medium was replaced with complete RPMI1640, and the cells were treated with 20 µg/mL of Nano-Ni. After 24 h Nano-Ni treatment, the cells were collected to determine the transfection efficiency and Rad52 expression.

### Soft agar colony formation assay

After treatment with 0.25 and 0.5 µg/mL of Nano-Ni as described above, cells were tested for their ability to grow in soft agar to evaluate their anchorage-independent growth. 2.5 × 10^5^ cells were plated in 5 mL of 0.33% agar in complete medium overlaid onto a solid layer of 0.5% agar in complete medium. After 6 weeks of growth in the incubator, the colonies were stained with INT/BCIP working solution [75 µL of INT/BCIP stock solution (Roche Diagnostics, Indianapolis, IN) in 10 mL of 0.1 M Tris-buffer, pH 9.5, 0.05 M MgCl_2_, and 0.1 M NaCl] as described in a previous study [56]. The images were captured by ChemiDoc XRS (Bio-Rad, Hercules, CA). The stained colonies with their areas greater than 0.1 mm^2^ were counted by NIH ImageJ Software (http://imagej.nih.gov/ij/).

### Exposure of mice to nickel nanoparticles

Animal use was reviewed and approved by the University of Louisville Institutional Animal Care and Use Committee. Eight-week-old male C57BL/6 J mice were obtained from The Jackson Laboratory (Bar Harbor, ME), and allowed to acclimatize for 1–2 weeks before Nano-Ni exposure. The *gpt* delta transgenic mice, which were in a C57BL/6 J background, were originally obtained from Dr. Takehiko Nohmi at the National Institute of Health Science in Japan [57], and bred in the animal facility of University of Louisville. The mice were housed in an air-conditioned room (temperature of 20 ± 2 °C, relative humidity of 60 ± 10%) with a 12-h light and 12-h dark cycle environment with free access to food and water. The mice were grouped randomly and instilled intratracheally with 50 µg per mouse of Nano-Ni as described previously [2, 3]. The control mice were instilled with physiological saline. The intratracheal instillation model is an easy and reliable method compared with an inhalation study and has been widely used to identify particle toxicity and to compare responses to different particle types. The C57BL/6 J mice were sacrificed at day 7 or day 42 after Nano-Ni instillation, while *gpt* delta transgenic mice were sacrificed at four months after exposure. At the endpoint of the experiment, the mouse was anesthetized by intraperitoneal injection of 300 mg/kg body weight of 2,2,2-tribromoethanol (Alfa Aesar, Heysham, England). Depth of anesthesia was determined by a lack of response to a toe pinch. The abdominal cavity was opened surgically, and the animal was sacrificed by cutting a major blood vessel in the abdomen. The left lungs were collected, immediately frozen in liquid nitrogen, and stored at − 80 °C for later isolation of protein, total RNA, or genomic DNA. The right lungs were fixed with 10% neutral buffered formalin, dehydrated stepwise through an ascending series of alcohol solutions, degreased in xylene, embedded in paraffin, sectioned at 5 μm by a microtome (Thermo Scientific, Rockford, IL), and stained with hematoxylin and eosin (HE) stains (Fisher Scientific, Fair Lawn, NJ) as described in our previous studies [2, 3] or for immunohistochemistry staining.

### Immunohistochemistry staining

Immunohistochemistry staining was used to evaluate the expression of Ki-67, PCNA, HIF-1α, and γ-H2AX in paraffin-embedded lung sections as described previously [49]. Briefly, lung sections were deparaffinized, hydrated, and incubated in 10 mM sodium citrate (pH 6.0) with 0.05% Tween-20 for 30 min at 95 °C for antigen retrieval. To inactivate endogenous peroxidase, the lung sections were immersed in 0.3% H_2_O_2_ in methanol for 30 min at room temperature. Non-specific binding of antibodies was blocked by incubating sections with blocking solution (3% BSA, 5% normal goat serum, and 0.3% Triton X-100 in 1 × PBS) for at least 30 min at room temperature. Lung sections were then incubated with primary antibody for Ki-67, PCNA, γH2AX, or HIF-1α overnight at 4 °C. After being washed, sections were subsequently incubated with biotinylated secondary antibody for 1 h, HRP-conjugated streptavidin for another 1 h, and 3, 3-diaminobenzidine (DAB) solution until desired stain intensity develops, with three-time washing between each step. Sections were counterstained with hematoxylin, mounted, and examined under a light microscope.

### Determination of *gpt* mutant frequency

The *gpt* delta transgenic mice carry about 80 copies of transgene, lambda EG10 DNA, on chromosome 17. The lambda EG10 DNA carries the *gpt* gene of *E. coli* [57]. The enzyme encoded by *gpt* gene, guanine phosphoribosyltransferase, catalyzes phosphoribosylation of guanine, which is the obligatory step for the incorporation of guanine to DNA. This enzyme also phosphoribosylates 6-thioguanine (6-TG), which is toxic to cells when it is incorporated into DNA, thus allowing the selection of *gpt* mutants by 6-TG. The *gpt* mutations in the genomic DNA of mouse lungs were detected as described previously [49, 57]. Lambda EG10 phages were rescued using Transpack Packaging Extract (Stratagene, LaJolla, CA) according to the manufacturer’s instruction. *E.coli* YG6020 expressing Cre recombinase (provided by Dr. Nohmi) was infected with the rescued phages and spread on M9 salt plates containing chloramphenicol (Cm) and 6-thioguanine (6-TG). The plate was incubated at 37 °C for 72 h, which enables selection of colonies harboring a plasmid carrying genes for chloramphenicol acetyltransferase (CAT) and a mutated *gpt*. Those (Cm + 6-TG)-resistant colonies, which contain a mutated *gpt*, were counted. The 6-TG-resistant phenotype of the colony was again confirmed by streaking *E.coli* cells on the (Cm + 6-TG) agar plate and the plate was incubated at 37 °C for 72 h. To obtain the total number of Cm-resistant colonies, *E.coli* YG6020 was infected with an aliquot of rescued phage suspension and spread on M9 salt plates containing chloramphenicol (Cm) only, but without 6-TG. The plate was also incubated at 37 °C for 72 h. Mutant frequency was calculated by dividing the number of colonies growing on (Cm + 6-TG) agar plate by the number on Cm agar plate as described previously [49, 57].

### Colony PCR and DNA sequencing analysis of *gpt* mutants

The coding region of the *gpt* gene is 456 bp, which is convenient for the identification of mutation by DNA sequencing. Colony PCR and DNA sequencing analysis of *gpt* mutants were performed as described previously [49]. Colony PCR was performed to amplify a 739 bp DNA fragment containing the mutated *gpt* gene in the (Cm + 6-TG)-resistant colonies. PCR was performed on a Mastercycler (Eppendorf, Hauppauge, NY) for 35 cycles, each cycle using sequentially 94 °C for 30 s, 58 °C for 30 s, and 72 °C for 1 min. The forward primer was 5′-TACCACTTTATCCCGCGTCAGG-3′ while the reverse primer was 5′-ACAGGGTTTCGCTCAGGTTTGC-3′. The amplified PCR products were checked by agarose gel electrophoresis and sent to DNA core facility at University of Louisville for sequencing. The sequencing primers were either 5′-GAGGCAGTGCGTAAAAAGAC-3′ or 5′-CTATTGTAACCCGCCTGAAG-3′ as described previously [49].

### Statistical analysis

SigmaPlot 13.0 software (Systat Software, Inc., San Jose, CA) was used for statistical analysis. Data were expressed as the mean ± SEM. Differences between two groups were analyzed by t-test. When there were more than two groups, one-way analysis of variance (ANOVA) followed by Dunnett’s post-hoc test was used for comparisons with the control. If there were two independent variables on a dependent variable, two-way ANOVA followed by Holm-Sidak post-hoc test was employed. If necessary, transformation of data was used to achieve normally distributed data before analysis. A difference was considered statistically significant when a p-value was less than 0.05.

## Results

### In vitro short-term Nano-Ni exposure

#### Cytotoxicity of metal nanoparticles on human bronchial epithelial cells

**Fig. 1:**
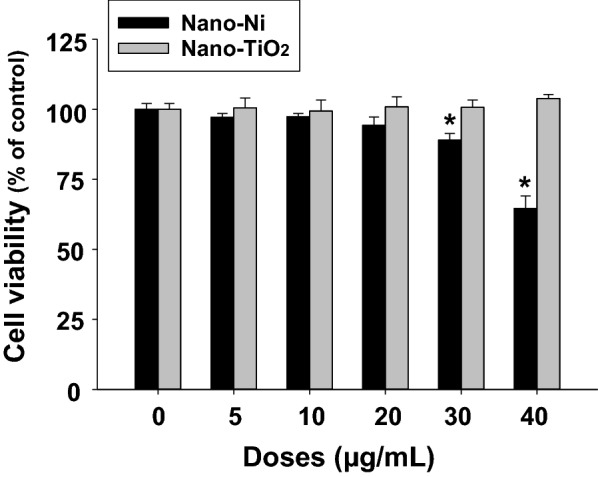
Cytotoxicity of metal nanoparticles on BEAS-2B cells. 5 × 10^3^ cells per well were seeded in 96-well plates. After overnight culture, cells were treated with different concentration of metal nanoparticles for 24 h. Cells without metal nanoparticle treatment were used as the control. Cytotoxicity was determined by MTS assay (Promega) and confirmed by alamarBlue™ assay (Invitrogen). Data are shown as mean ± SEM (n = 5 ~ 6). *, p < 0.05 vs. control

Exposure of normal human bronchial epithelial cells BEAS-2B to Nano-Ni at concentrations up to 20 µg/mL for 24 h did not cause significant cytotoxicity by MTS assay (Fig. 1), which detects the number of metabolically active cells from the extent that dehydrogenase enzymes convert a tetrazolium compound (MTS) into an aqueous, soluble, and colored formazan. However, exposure of the cells to 30 µg/mL and beyond of Nano-Ni caused significant cytotoxicity (Fig. 1). Nano-TiO_2_ exposure did not cause significant cytotoxicity on BEAS-2B cells at any of the investigated doses up to 40 µg/mL (Fig. 1). The results were further confirmed by alamarBlue™ assay (data not shown), which quantitatively measures the proliferation of cells by using the reducing power of living cells through an oxidation–reduction indicator.

#### Nano-Ni exposure caused DNA damage and DNA damage responses

**Fig. 2:**
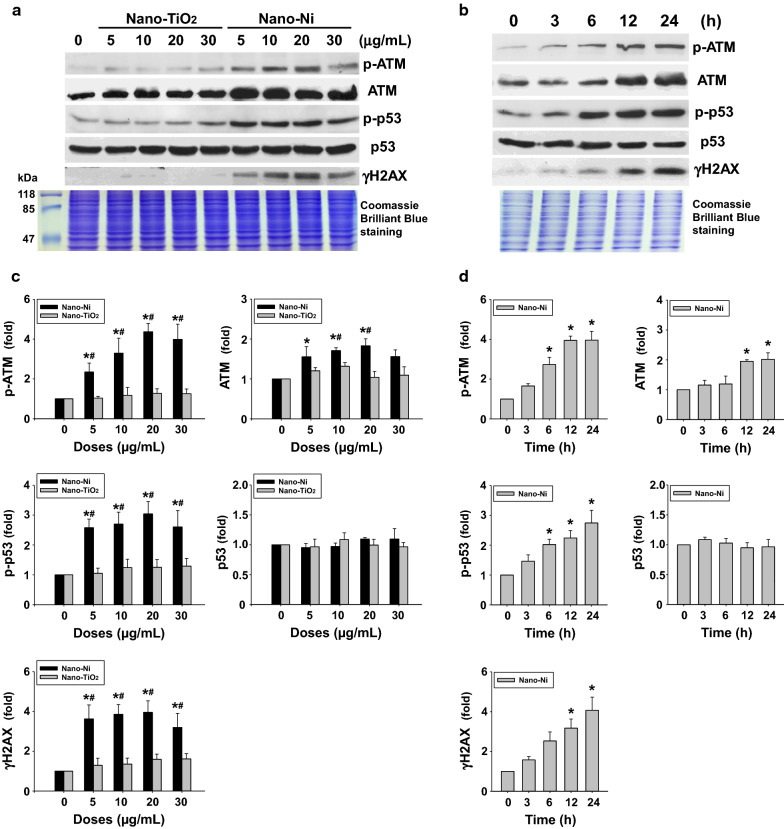
Nano-Ni exposure caused increased expression of DNA damage response-associated proteins in BEAS-2B cells (dose- and time-response studies). For the dose–response study, cells were treated with 5, 10, 20, and 30 µg/mL of Nano-Ni or Nano-TiO_2_ for 24 h. For the time-response study, cells were treated with 20 µg/mL of Nano-Ni for 3, 6, 12, and 24 h. Cells without treatment were used as the control. Nuclear protein was subjected to Western blot. Equal nuclear protein loading was verified by Coomassie Brilliant Blue staining. **A**, **B** are results of a single Western blot experiment, while **C**, **D** are quantified band densitometry readings averaged from at least 3 independent experiments ± SEM of Western blot results. * p < 0.05 vs. control; # p < 0.05 vs. same dose of Nano-TiO_2_-treated group. p-ATM, phosphorylated ATM at Ser1981; p-p53, phosphorylated p53 at Ser15

Expression of DNA damage response (DDR)-associated proteins in BEAS-2B cells after metal nanoparticle exposure was determined by Western blot. Our results showed that Nano-Ni, but not Nano-TiO_2_, significantly upregulated phosphorylated ATM at Ser1981 (p-ATM) in a dose- and a time-dependent manner (Fig. 2). The expression of total ATM also increased after Nano-Ni, but not Nano-TiO_2_, exposure (Fig. 2). A dose- and a time-dependent increase of phosphorylated p53 at Ser15 (p-p53) was also observed after Nano-Ni exposure, while the expression of total p53 was not affected by Nano-Ni (Fig. 2). Phosphorylation of H2AX (γH2AX) is a sensitive marker for DNA double strand breaks (DSBs), which will accumulate at the sites of DNA DSBs [20]. Our results demonstrated increased γH2AX expression in BEAS-2B cells exposed to Nano-Ni (Fig. 2), indicating Nano-Ni caused DNA DSBs in BEAS-2B cells. However, Nano-TiO_2_ exposure did not cause statistically significant increase in the p-p53 and γH2AX (Fig. 2).

#### Nano-Ni exposure induced HIF-1α nuclear accumulation, miR-210 up-regulation, and Rad52 down-regulation

**Fig. 3:**
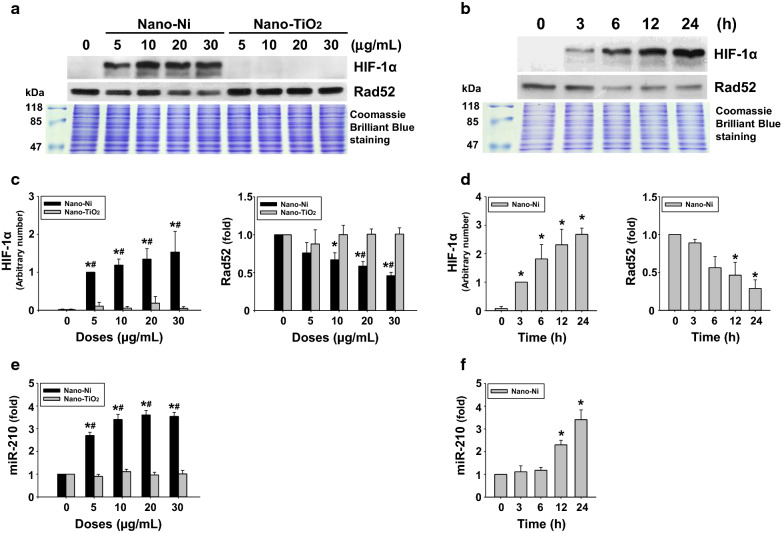
Nano-Ni up-regulated HIF-1α and miR-210 and down-regulated Rad52 in BEAS-2B cells (dose- and time-response studies). For the dose–response study, cells were treated with 5, 10, 20, and 30 µg/mL of Nano-Ni or Nano-TiO_2_ for 24 h. For the time-response study, cells were treated with 20 µg/mL of Nano-Ni for 3, 6, 12, and 24 h. Cells without treatment were used as the control. **A**, **B** are results of a single Western blot experiment. Nuclear protein was subjected to Western blot. Equal nuclear protein loading was verified by Coomassie Brilliant Blue staining. **C**, **D** are quantified band densitometry readings averaged from at least 3 independent experiments ± SEM of Western blot results. **E**, **F** miR-210 expression was determined by real-time PCR. Values of miR-210 expression were normalized to the endogenous control U6 snRNA. Data are shown as mean ± SEM (n = 3). * p < 0.05 vs. control; # p < 0.05 vs. same dose of Nano-TiO_2_-treated group

In the control BEAS-2B cells, only very faint or no HIF-1α expression was observed by Western blot. However, exposure of the cells to as low as 5 µg/mL of Nano-Ni for 24 h or 20 µg/mL of Nano-Ni for as early as 3 h caused significant HIF-1α nuclear accumulation (Fig. 3a–d). Equal doses of Nano-TiO_2_ exposure did not cause HIF-1α nuclear accumulation (Fig. 3a, c). Rad52 is a DNA repair protein functioned in homologous recombination repair (HRR) in mammalian cells [58, 59]. Nano-Ni, but not Nano-TiO_2_, exposure significantly down-regulated Rad52 expression when BEAS-2B cells were exposed to 10, 20, and 30 µg/mL of Nano-Ni for 24 h or to 20 µg/mL of Nano-Ni for 12 or 24 h (Fig. 3a–d). Moreover, miR-210, a hypoxamiR, was up-regulated by Nano-Ni exposure, which was observed after cells were exposed to 5, 10, 20, and 30 µg/mL of Nano-Ni for 24 h (Fig. 3e) or 20 µg/mL of Nano-Ni for 12 and 24 h (Fig. 3f).

#### Inhibition of and/or knocking-out HIF-1α or miR-210 ameliorated Nano-Ni-induced Rad52 down-regulation

**Fig. 4:**
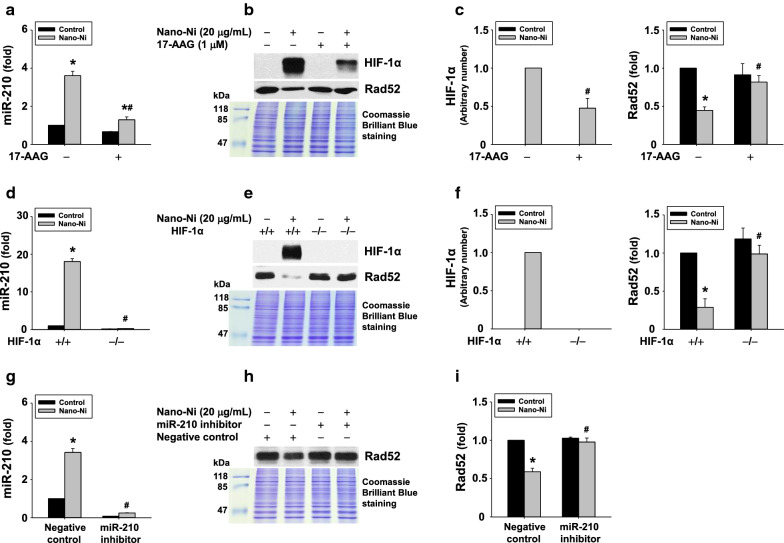
Inhibition of and/or knocking-out HIF-1α or miR-210 abolished Nano-Ni-induced Rad52 down-regulation. **A**–**C** BEAS-2B cells were pretreated with 1 µM of 17-AAG for 4 h, followed by treatment with 20 µg/mL of Nano-Ni for 24 h. **D**–**F** HIF-1α wild-type (+ / +) and knock-out (−/−) cells were treated with 20 µg/mL of Nano-Ni for 24 h. **G**–**I** BEAS-2B cells were transfected with mirVana™ miRNA inhibitor for has-miR-210-3p or Negative Control #1 for 24 h, followed by treatment with 20 µg/mL of Nano-Ni for another 24 h. **A**, **D**, **G** miR-210 expression was determined by real-time PCR. Values of miR-210 expression was normalized to the endogenous control U6 snRNA. Data are shown as mean ± SEM (n = 3–4). **B**–**C**, **E**–**F**, **H**–**I** Nuclear proteins were subjected to Western blot. Equal nuclear protein loading was verified by Coomassie Brilliant Blue staining. **B**, **E**, **H** are results of a single Western blot experiment, while **C**, **F**, **I** are quantified band densitometry readings averaged from at least 3 independent experiments ± SEM of Western blot results. *, p < 0.05 vs. control; #, p < 0.05 vs. group with Nano-Ni treatment, but without 17-AAG treatment (**A**, **C**), Nano-Ni-treated HIF-1α (+ / +) group (**D**, **F**), or group with Negative Control transfection and Nano-Ni treatment (**G**, **I**)

To explore whether Nano-Ni-induced HIF-1α nuclear accumulation was involved in Nano-Ni-induced miR-210 up-regulation and Rad52 down-regulation, a Hsp90 inhibitor, 17-AAG, was used to promote HIF-1α degradation and diminish its transcriptional activity, thus preventing its nuclear accumulation [51]. Our results showed that pretreatment of cells with 1 µM of 17-AAG significantly abolished Nano-Ni-induced miR-210 up-regulation (Fig. 4a) and Rad52 down-regulation (Fig. 4b, c), suggesting the involvement of Nano-Ni-induced HIF-1α nuclear accumulation in Nano-Ni-induced miR-210 up-regulation and Rad52 down-regulation. The results were further confirmed by using HIF-1α knock-out cells; Nano-Ni exposure did not elicit miR-210 up-regulation (Fig. 4d) and Rad52 down-regulation (Fig. 4e, f) in HIF-1α knock-out cells.

To observe whether Nano-Ni-induced miR-210 up-regulation was involved in Nano-Ni-induced Rad52 down-regulation, BEAS-2B cells were transfected with mirVana™ miRNA inhibitor against has-miR-210-3p. Cells transfected with inhibitor Negative Control #1 were used to observe if there are any “off-target” effects. Our results showed that inhibition of miR-210 expression restored Nano-Ni-induced Rad52 down-regulation (Fig. 4g–i).

On the other hand, to investigate whether Nano-Ni-induced HIF-1α nuclear accumulation was involved in Nano-Ni-induced up-regulation of DNA damage response (DDR)-associated proteins, Western blot was used after cells were pretreated with 1 µM of 17-AAG for 4 h followed by 20 µg/mL of Nano-Ni for 24 h. The results showed that inhibition of HIF-1α did not affect Nano-Ni-induced increased expression of DDR-associated proteins such as phosphorylated ATM at Ser1981 (p-ATM), phosphorylated p53 at Ser15 (p-p53), and γH2AX (Additional file 1a). The HIF-1α knock-out cells were introduced to confirm the results; Nano-Ni exposure caused up-regulation of DDR-associated proteins in both HIF-1α wild-type (+ / +) and knock-out (−/−) cells (Additional file 1b), suggesting Nano-Ni-induced HIF-1α nuclear accumulation is not involved in Nano-Ni-induced up-regulation of DDR-associated proteins.

### In vitro long-term Nano-Ni exposure

#### Long-term Nano-Ni exposure caused DNA damage and dysregulation of HIF-1α/miR-210/Rad52 pathway

**Fig. 5:**
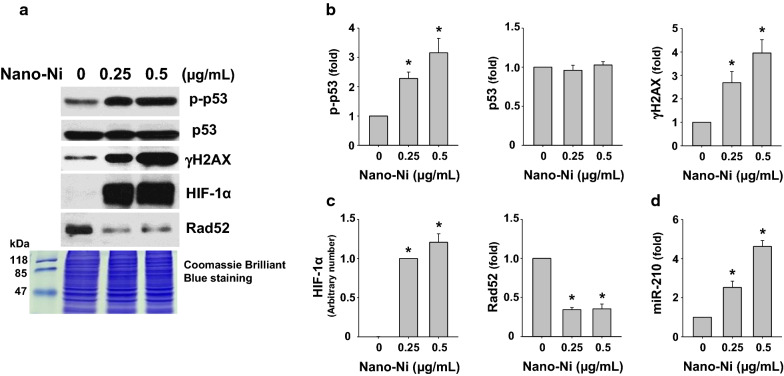
Dysregulation of DNA damage response-associated proteins and the HIF-1α/miR-210/Rad52 signaling pathway in BEAS-2B cells after long-term Nano-Ni exposure. BEAS-2B cells were treated with 0, 0.25 and 0.5 µg/mL of Nano-Ni for 21 cycles as described in the Methods. **A** is the result of a single Western blot experiment. Nuclear protein was subjected to Western blot. Equal nuclear protein loading was verified by Coomassie Brilliant Blue staining. **B**, **C** are quantified band densitometry readings averaged from 3 independent experiments ± SEM of Western blot results. **D** miR-210 expression was determined by real-time PCR. Values of miR-210 expression was normalized to the endogenous control U6 snRNA. Data are shown as mean ± SEM (n = 3). *, p < 0.05 vs. control

BEAS-2B cells were treated with low doses (0.25 and 0.5 µg/mL) of Nano-Ni for 21 cycles as described in the Methods, and the expression of DNA damage response-associated proteins, HIF-1α, and Rad52 were assessed by Western blot, while the miR-210 expression level was determined by real-time PCR. Our results demonstrated that long-term low doses of Nano-Ni treatment caused DNA damage, which was reflected by increased phosphorylation of DNA damage response-associated proteins such as phosphorylated p53 at Ser15 and γH2AX (Fig. 5a, b). Long-term low doses of Nano-Ni exposure also caused HIF-1α nuclear accumulation (Fig. 5a, c), Rad52 down-regulation (Fig. 5a, c), and miR-210 up-regulation (Fig. 5d) in BEAS-2B cells.

#### Long-term Nano-Ni exposure caused cell transformation and overexpression of Rad52 attenuated Nano-Ni-induced cell transformation

**Fig. 6:**
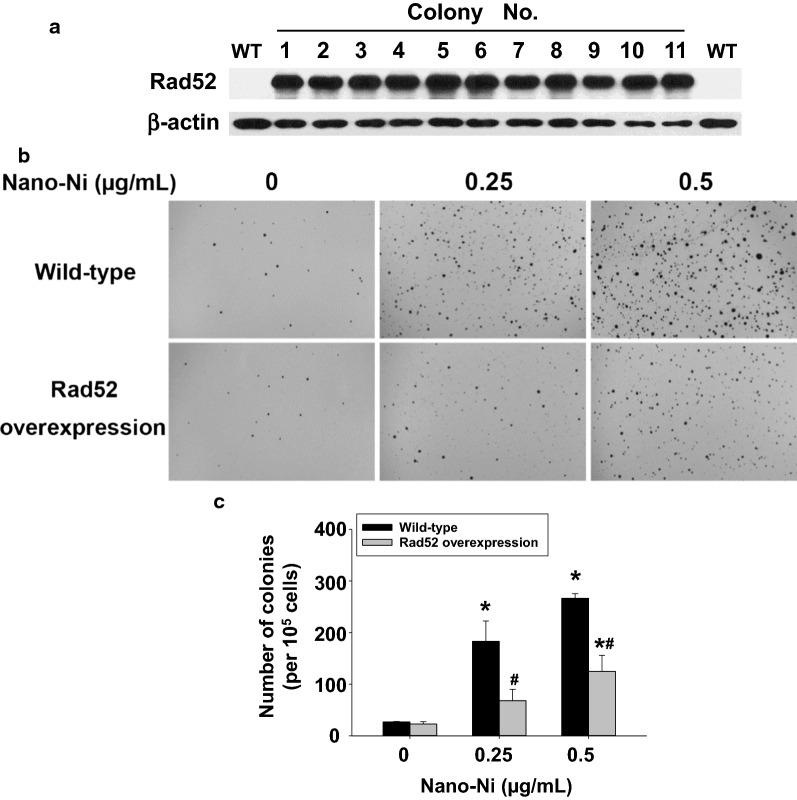
Long-term Nano-Ni exposure induced cell transformation, which was attenuated by overexpression of Rad52. **A** BEAS-2B cells were transduced with lentiviral particles containing human Rad52 ORF as described in the Methods. 11 puromycin-resistant colonies were picked and expanded, and the total protein from each colony was isolated for Western blot. The exposure time was 2 s. β-actin served as loading control. WT, wild-type. **B**, **C** The effects of Nano-Ni on anchorage-independent growth of cells by soft agar colony formation assay. Cells were exposed to 0, 0.25 and 0.5 µg/mL of Nano-Ni for 21 cycles as described in the Methods. The colonies were stained with INT/BCIP solution (**B**) and quantified using ImageJ software (**C**). Data are shown as mean ± SEM (n = 3 ~ 6). *, p < 0.05 vs. control; #, p < 0.05 vs. wild-type group with same dose of Nano-Ni treatment

After long-term low doses (0.25 and 0.5 µg/mL) of Nano-Ni exposure, soft agar colony formation assay was performed to observe the ability of anchorage-independent growth of cells. The colonies grew in the soft agar were counted by using ImageJ software after INT/BCIP staining. Our results showed that long-term (21 cycles, ~ 150 days) Nano-Ni exposure resulted in significant increase in the number and areas of colonies in the soft agar (Fig. 6b, c), suggesting that long-term Nano-Ni exposure can cause cells from normal to malignant transformation. However, we did not observe increased colony formation when the cells were exposed to Nano-Ni for 10 cycles (~ 73 days) (data not shown). Since DNA repair protein Rad52 was significantly down-regulated after Nano-Ni exposure, this raises the question whether Rad52 down-regulation is involved in Nano-Ni-induced cell transformation. Thus, we transduced cells with lentiviral particles containing ORF of human Rad52. After puromycin selection, 11 colonies were picked and expanded, and Western blot was used to confirm Rad52 overexpression in these colonies (Fig. 6a). Since Rad52 expression was significantly increased after transduction, the endogenous Rad52 expression in the wild-type (WT) cells could not be observed when the exposure time was 2 s (Fig. 6a), however, which could be observed when the exposure time was extended to 1 min (Additional file 2). The colony No. 1 was selected for long-term low doses of Nano-Ni exposure. Our results showed that overexpression of Rad52 significantly reduced Nano-Ni-induced cell transformation (Fig. 6b, c).

### In vivo Nano-Ni exposure

#### Exposure of mice to Nano-Ni caused up-regulation of γH2AX and dysregulation of HIF-1α/miR-210/Rad52 pathway

**Fig. 7:**
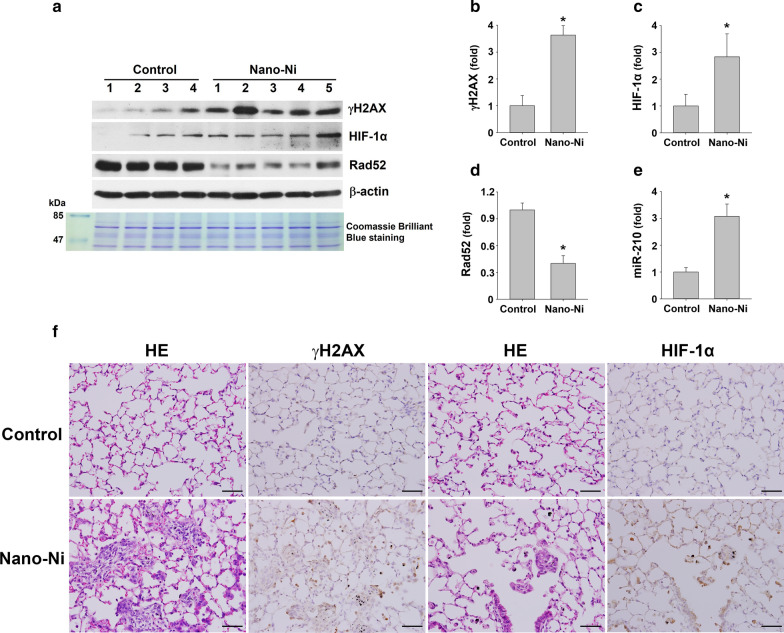
HIF-1α/miR-210/Rad52 signaling and expression of DNA damage response protein in mouse lungs after Nano-Ni exposure. Mice were instilled intratracheally with 50 µg per mouse of Nano-Ni. Control mice were instilled with physiological saline. Lung tissues were collected at day 7 after Nano-Ni exposure. **A** is the results of Western blot experiment, while **B**–**D** are results quantified by ImageJ software and normalized by internal control β-actin. **E** miR-210 expression was determined by real-time PCR. Values of miR-210 expression was normalized to the endogenous control U6 snRNA. Data are shown as mean ± SEM (n = 4–5). *, p < 0.05 vs. control. **F** Expression of HIF-1α and γH2AX in mouse lungs by immunohistochemical staining. Increased number of HIF-1α and γH2AX positive cells (brown staining) were observed in the mouse lungs after Nano-Ni exposure. Scale bars represent 50 µm for all panels

Since Nano-Ni-induced DNA damage and DNA damage responses were observed in BEAS-2B cells, we continued to investigate whether Nano-Ni could also cause similar effects in vivo. C57BL/6J mice were intratracheally instilled with 50 µg per mouse of Nano-Ni and mouse lungs were collected at day 7 after exposure. The expression level of DNA damage-associated protein γH2AX was determined by Western blot and immunohistochemistry staining. Our results showed that Nano-Ni exposure caused significant up-regulation of γH2AX (Fig. 7a, b, f), indicating DNA damage in mouse lungs after Nano-Ni instillation.

The dysregulation of HIF-1α/miR-210/Rad52 pathway was also examined in mouse lungs after Nano-Ni exposure by Western blot and real-time PCR. Our results showed that Nano-Ni exposure caused significant up-regulation of HIF-1α (Fig. 7a, c, f) and miR-210 (Fig. 7e), while Rad52 expression was down-regulated (Fig. 7a, d), suggesting dysregulation of HIF-1α/miR-210/Rad52 pathway in mouse lungs after Nano-Ni exposure. Nuclear accumulation of HIF-1α in the cells of mouse lungs was further confirmed by immunohistochemistry staining (Fig. 7f).

#### Nano-Ni exposure induced cell proliferation in mouse Iungs

**Fig. 8:**
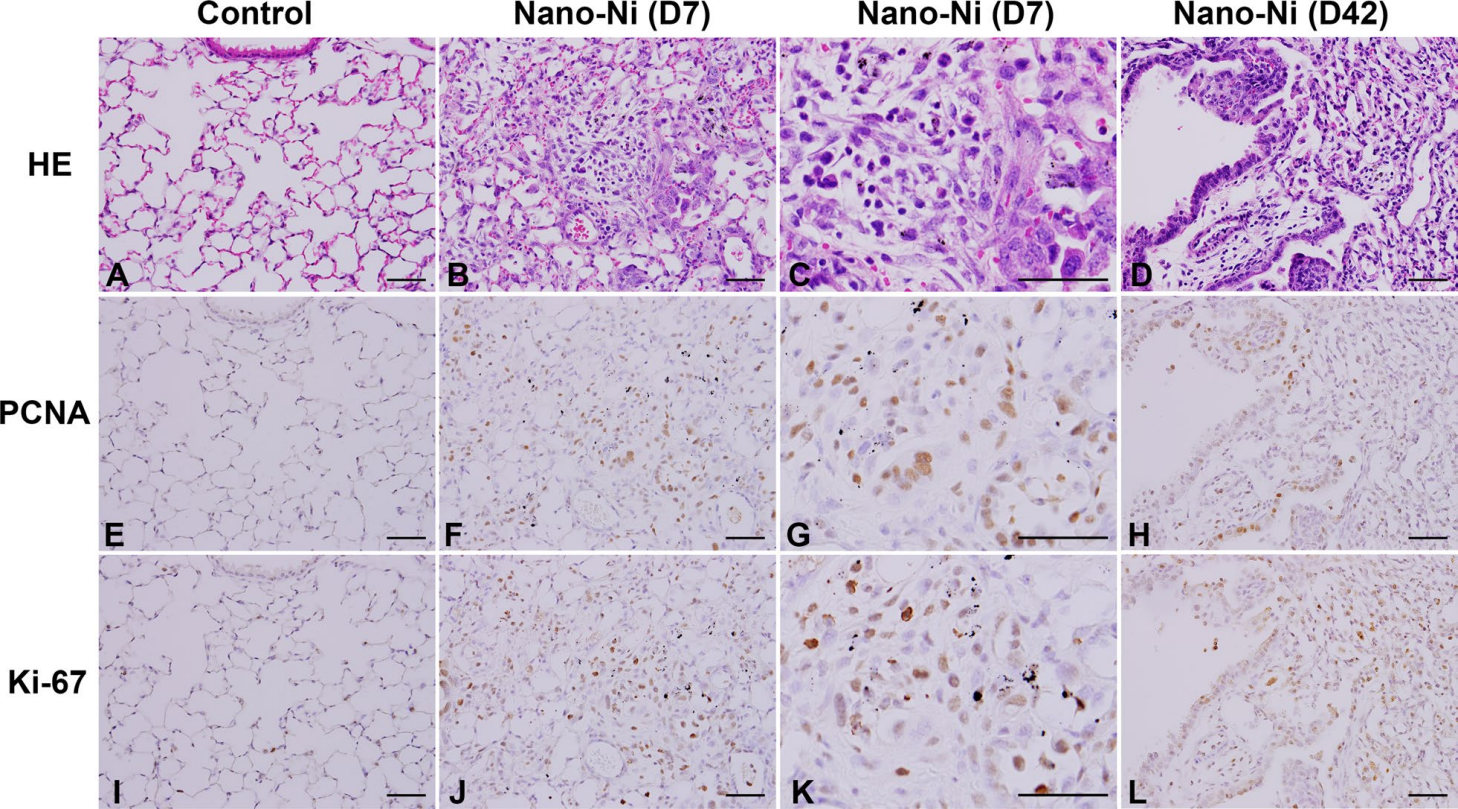
Increased number of PCNA- and Ki-67-positive cells in mouse lungs after Nano-Ni exposure by immunohistochemical staining. Mice were instilled intratracheally with 50 µg per mouse of Nano-Ni. Control mice were instilled with physiological saline. Lung tissues were collected at day 7 (D7) and day 42 (D42) after Nano-Ni exposure. A, E, and I show the normal structure of lung parenchyma in a control mouse. Increased number of PCNA (F–H) and Ki-67 (J-L) positive cells (brown staining) were observed in the mouse lungs after Nano-Ni exposure. Scale bars represent 50 µm for all panels

To determine whether Nano-Ni exposure caused cell proliferation, immunohistochemistry staining was performed on lung sections obtained from mice at day 7 and day 42 after Nano-Ni exposure by using anti-Ki-67 and anti-PCNA antibodies. Our results showed increased number of Ki-67-positive and PCNA-positive cells in Nano-Ni-instilled mouse lungs as compared to that in the control lungs at day 7 after instillation (Fig. 8), suggesting cell proliferation in mouse lungs after Nano-Ni exposure. Although the number of proliferating cells decreased at day 42 as compared to day 7 after Nano-Ni exposure, mouse lungs still exhibited a significantly increased number of Ki-67-positive and PCNA-positive cells (Fig. 8).

#### Nano-Ni exposure did not cause increased mutant frequency (MF) in genomic DNA of mouse lungs

Our in vitro results have demonstrated that long-term low doses of Nano-Ni exposure caused BEAS-2B cells to undergo normal to malignant transformation. To explore whether Nano-Ni exposure caused increased mutant frequency and the mutation spectrum, *gpt* delta transgenic mouse model was employed. The coding region of *gpt* gene is only 456 bp, which is convenient for the identification of mutation by DNA sequencing [49, 57]. *gpt* delta transgenic mice were intratracheally instilled with 50 µg per mouse of Nano-Ni, while control mice were instilled with physiological saline. After four months, the mouse lungs were collected, and the genomic DNA was isolated. *gpt* mutant frequency and mutation spectrum were determined as described in the Methods. Our results showed that the mutant frequency in Nano-Ni-instilled lungs was 11.6 ± 1.8 (mean ± SEM, ×10^−6^), which was similar to that in the control lungs (10.6 ± 3.6) (mean ± SEM, ×10^−6^) (Table 1 & Additional file 3). The mutation spectrum in the Nano-Ni-instilled mice was also similar to those in the control mice (Table 2). These results suggest that Nano-Ni exposure did not cause increased *gpt* mutant frequency and some kinds of DNA mutations, such as base substitution and simple and small base insertions/deletions, are not the main types of Nano-Ni-induced DNA damage.

**Table 1:** Mutant frequency (MF) of *gpt* gene in mouse lungs

Treatment	Sex	No. of rescued colonies	No. of mutants	MF (× 10^−6^)	Average of MF ± SEM (× 10^−6^)
Control	F	477,000	5	10.5	
	F	540,000	9	16.7	
	M	229,500	2	8.7	
	M	1,429,500	15	10.5	11.6 ± 1.8
Nano-Ni	F	697,500	3	4.3	
	F	529,500	4	7.6	
	M	369,000	9	24.4	
	M	844,500	9	10.7	
	M	2,004,000	12	6.0	10.6 ± 3.6

*gpt* delta transgenic mice were instilled intratracheally with 50 µg per mouse of Nano-Ni. Control mice were instilled with physiological saline. Lung tissues were collected at four months after instillation

**Table 2:** Summary of *gpt* mutations in mouse lungs

Type of mutation in *gpt*	Control		Nano-Ni	
	No.	%	No.	%
Transition				
G:C to A:T	6	31.6	9	33.3
A:T to G:C	2	10.5	2	7.4
Transversion				
G:C to T:A	6	31.6	8	29.6
G:C to C:G	0	0	0	0
A:T to T:A	0	0	1	3.7
A:T to C:G	2	10.5	2	7.4
Deletions	1	5.3	1	3.7
Insertions	2	10.5	3	11.1
Others	0	0	1	3.7
Total	19	100	27	100

## Discussion

Nano-Ni belongs to the important class of transition metal nanoparticles and has found a wide range of applications due to its unique chemical and physical properties. As the use of Nano-Ni continues to expand, the risk of occupational and non-occupational exposure to Nano-Ni is increasing. Nano-Ni may be genotoxic and carcinogenic because of the chemical nature of the native metal. Thus, it is important to understand the toxic effects, especially the genotoxic and carcinogenic effects, of nickel nanoparticles and the potential underlying mechanisms.

**Fig. 9:**
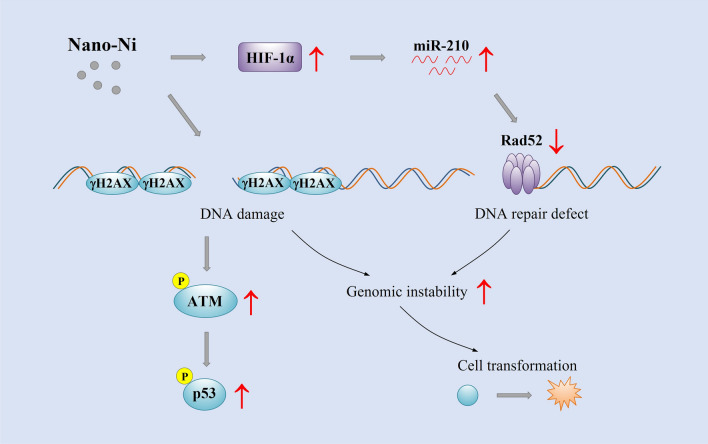
Schematic diagram of the possible mechanisms involved in Nano-Ni-induced cell transformation. Nano-Ni exposure causes DNA damage, which induces the DNA damage response. Repeated insults may cause erroneous DNA repair. Nano-Ni exposure also induces HIF-1α nuclear accumulation, which causes defective DNA repair through up-regulation of miR-210 and down-regulation of Rad52. Both DNA damage and defective DNA repair may contribute to increased genomic instability, leading to cell transformation

DNA damage, which includes base mismatches, insertions/deletions, single-strand DNA breaks (SSBs), double-strand DNA breaks (DSBs), DNA adducts, etc., occurs constantly through a series of exogenous and endogenous insults [15, 16]. In this study, Nano-Ni-induced DNA damage was first determined in vitro by using BEAS-2B cells, and then in vivo by intratracheal instillation of Nano-Ni into mice. Since DNA damage elicits a series of DNA damage responses (DDR) to occur, we examined DDR-associated proteins, such as ATM, p53, and H2AX, to observe whether Nano-Ni exposure induced DNA damage (Fig. 9). Our previous studies have shown that ATM, p53, and γH2AX are sensitive markers for Nano-Co-induced DNA damage [18, 49]. ATM primary responds to DNA DSBs and activated ATM modifies directly or indirectly a broad range of targets including p53 and H2AX [17]. Phosphorylation of H2AX (γH2AX) is a key step in signaling and initiating the repair of DSBs, thus γH2AX has been verified as a sensitive marker for DSBs [60, 61]. Our results demonstrated that exposure of normal human bronchial epithelial cells BEAS-2B to Nano-Ni caused increased phosphorylation of ATM at Ser1981, p53 at Ser15, and histone H2AX, indicating Nano-Ni exposure caused DSBs in BEAS-2B cells. Nano-Ni-induced DNA damage was also observed in vivo; increased expression of γH2AX was observed in mouse lungs after mice were intratracheally instilled with Nano-Ni.

Nano-Ni-induced DNA damage has been observed in previous studies. By using comet assay, increased DNA damage were observed after Nano-Ni exposure in human bronchial epithelial cells HBEC [62], human skin epidermal cells A431 [63], adenocarcinomic human type II alveolar epithelial cells A549 [64], human breast carcinoma cells MCF-7 [65], and wild‐type Chinese hamster lung fibroblasts V79 [66]. However, it is unclear whether the observed DNA damage are single-strand breaks (SSBs) or double-strand breaks (DSBs) since comet assay can detect both. Our results demonstrated that Nano-Ni exposure resulted in DNA DSBs, which was reflected by the increased expression of γH2AX, a sensitive marker for DSBs [60, 61]. Although a previous study failed to detect an increase in γH2AX fluorescence by flow cytometry in HBEC cells after Nano-Ni exposure [62], different cell lines, different doses of Nano-Ni, different physical and chemical properties of Nano-Ni, and different methods used to detect the alteration of γH2AX expression may all contribute to the variable results observed.

The mechanisms underlying Nano-Ni-induced DNA damage have not been clearly elucidated. Although oxidative stress has been suggested to be an important underlying mechanism in different in vitro model systems including HBEC [62], A431 [63], and MCF-7 [65], no intracellular ROS was detected in A549 cells after Nano-Ni exposure [67]. We also did not detect any increased ROS generation in BEAS-2B cells after Nano-Ni exposure by using 2’,7’-dichlorodihydrofluorescin diacetate (H2DCF-DA) method (data not shown). Nano-Ni and their released nickel ions may directly target DNA to cause its damage. Because of their small sizes, Nano-Ni may pass directly through cell membrane and nuclear membrane to reach cell nucleus. In fact, we have observed previously Nano-Ni-phagocytized macrophages in mouse lungs and bronchioalveolar lavage fluid (BALF) after Nano-Ni instillation by microscope, where the Nano-Ni are aggregated [2, 3, 68]. Aggregated Nano-Ni were also observed in alveolar septa, pneumocytes, lymphocytes, etc. in mouse lungs [2]. Latvala et al. also found cellular uptake of nickel nanoparticles in human alveolar epithelial cells A549 by TEM [67]. Nano-Ni may also cause indirect DNA damage due to inhibition of DNA repair, which was confirmed in this study; we found decreased expression of Rad52, a homologous recombinational repair (HRR) gene, in Nano-Ni-exposed BEAS-2B cells and Nano-Ni-instilled mouse lungs. The mechanistic understanding of Nano-Ni-induced DNA damage is still in its infancy and needs to be further explored.

In this study, we also demonstrated that Nano-Ni exposure caused dysregulation of HIF-1α/miR-210/Rad52 pathway (Fig. 9). Nano-Ni exposure induced significant HIF-1α nuclear accumulation, which is consistent with our and other previous reports; Nano-Ni-induced HIF-1α nuclear accumulation has been observed in multiple cell models, such as human lung epithelial cells H460 [69], human monocytes U937 [9], and human epidermal keratinocytes HaCaT [10]. Although how Nano-Ni induces HIF-1α nuclear accumulation is still unclear, nickel has been reported to be able to inactivate prolyl hydroxylases (PHDs) by depleting intracellular ascorbate [26, 70, 71], substituting for Fe^2+^ in the regulatory dioxygenases including PHDs, or suppressing the delivery of Fe^2+^ into cells by binding more tightly than Fe^2+^ to the membrane transporter DMT-1 [26, 72], resulting in hypoxia-like stress.

miR-210 is a known hypoxamiR and has been shown to have a hypoxia-responsive element (HRE) on its promoter, indicating miR-210 is a direct HIF-1α target [37]. We found here that expression of miR-210 increased after Nano-Ni exposure, which was through Nano-Ni-induced HIF-1α nuclear accumulation; targeting HIF-1α using a Hsp90 inhibitor, 17-AAG, or knocking-out HIF-1α abolished Nano-Ni-induced miR-210 up-regulation. Inhibition of Hsp90, a molecular chaperone, causes O_2_/PHD/VHL-independent proteasomal degradation of HIF-1α and diminishes HIF-1α transcriptional activity [51]. Rad52 was identified as a miR-210 target; forced expression of miR-210 was able to suppress Rad52 [40, 48].

Rad52 has critical roles in homologous recombination (HR) repair of DSBs and restarting stalled or collapsed replication forks, thus playing an important role in cellular response to DNA damage and the control of genomic integrity [58, 59]. Nickel exposure has been found to affect DNA repair genes. A previous study has shown that nickel and arsenite inhibit the repair of radiation-induced DNA double-strand breaks (DSBs) [73]. Down-regulation of DNA repair genes have been verified in peripheral blood mononuclear cells of nickel refinery workers [74]. Nickel exposure leads to down-regulation of DNA repair proteins involved in homology-dependent DNA DSB repair (HDR) and mismatch repair (MMR) in tumorigenic and non-tumorigenic human lung cells [75]. In this study, we found that Nano-Ni exposure caused down-regulation of Rad52, which was through up-regulation of miR-210 induced by HIF-1α activation, since inhibition of and/or knocking-out HIF-1α or miR-210 ameliorated Nano-Ni-induced Rad52 down-regulation.

In the current study, we found that long-term (21 cycles, ~ 150 days) low doses of Nano-Ni exposure caused normal human bronchial epithelial cells BEAS-2B to undergo malignant transformation. Previous studies showed controversial results on whether Nano-Ni can cause cell transformation. One study showed that both metallic nickel nano- and fine particles increased anchorage-independent colony formation in mouse epidermal JB6 P + cells in the soft agar assay [76]. However, Gliga et al. did not observe the ability of nickel-containing nanoparticles to transform BEAS-2B cells after the cells were exposed to 0.5 μg/mL of nickel-containing nanoparticles for 6 weeks [77], although they did observe more colonies in the soft agar in the nickel-containing nanoparticles groups. The main reason underlying the different results is the different exposure time (6 weeks vs. 150 days) since we also did not observe increased colony formation when the cells were exposed to Nano-Ni for 10 cycles (73 days). 6-week exposure may be not long enough for the cells to accumulate genetic changes to cause cell transformation. Other factors such as exposure dose, exposure method, different manufacturers of the particles, etc. also cannot be completely excluded. We also observed that restoration of Rad52 level by transducting lentiviral particles containing human Rad52 ORF into the cells significantly reduced Nano-Ni-induced cell transformation, indicating defects in homologous recombination repair of DSBs is involved in Nano-Ni-induced cell transformation.

We observed DNA damage and DNA repair defects in our in vivo studies by intratracheally instillation of Nano-Ni into mice. However, by using *gpt* transgenic mouse model, we did not observe certain kinds of DNA mutations, including base substitution and simple and small base insertions/deletions, suggesting that they may not be involved in cell transformation after Nano-Ni exposure. No increased mutant frequency in the *gpt* gene was observed and the mutation profile in the lungs of Nano-Ni-instilled mice was similar to that of the controls. Previous studies also demonstrated that after Nano-Ni exposure, no increased HPRT mutant frequency was observed in human bronchial epithelial cells HBEC [62] and wild‐type Chinese hamster lung fibroblasts V79 [66].

Increased expression of proliferation markers such as Ki-67 and PCNA was observed in mouse lungs after Nano-Ni exposure. Ki-67 is a nuclear protein which is only found in proliferating cells and absent in resting cells, thus making it an excellent marker for proliferating cells and determining the growth fraction of a given cell population [78]. Ki-67 protein is strongly associated with tumor cell proliferation and is an established prognostic marker for the assessment of biopsies from patients with cancer [78, 79]. PCNA is an important hub protein and central to both DNA replication and repair [80, 81]. PCNA forms a ring around the DNA to facilitate and control DNA replication. Thus, immunohistochemical staining of PCNA detects not only actively dividing cells, but also those in the process of DNA repair [80, 81]. It is well known that genetic alterations that lead to cancer are more likely to occur in actively proliferating tissues. Cells with high rates of proliferation are more susceptible to DNA damage and tumorigenesis [82, 83]. Our present study clearly showed that Nano-Ni exposure caused not only DNA damage, but also cell proliferation. The proliferating cells may be more sensitive to Nano-Ni, which may damage DNA further.

Replacing, reducing, and refining (3Rs) the use of in vivo experimentation is important and necessary, especially in the field of nanomaterials [84]. The toxicity of nanoparticles is dependent on their sizes and physico-chemical properties; thus each property variant of nanoparticles may be required to assess its toxicity, which is both expensive and time-consuming to investigate on animals. Although there are many in vitro methods to assess genotoxicity of nanoparticles such as comet assay, micronuclei assay, etc., only in vivo studies can assess the pulmonary effects of nanoparticles since validated alternatives to in vivo pulmonary toxicity tests are not currently available [85]. Moreover, because of their small sizes, nanoparticles are not as readily phagocytized by macrophages as larger particles. They consequently can penetrate much more rapidly through the epithelium and reach the endothelium. They may even enter the blood circulation, resulting in their translocation to other organs. These responses result from the complex interactions of multiple cell types, including epithelial cells, endothelial cells, inflammatory cells, and fibroblasts. There is no way currently to fully replicate these interactions except in an animal model. We previously determined the effects of Nano-Ni on mouse lungs [2, 3] and on mouse peripheral blood monocytes [8]. Here, we investigated the effects of Nano-Ni on DNA damage and DNA repair pathways in mouse lungs.

In this study, Nano-TiO_2_ was used as a negative control since our previous studies have shown that exposure to Nano-TiO_2_ did not induce HIF-1α nuclear accumulation in human monocytes U937 [9] or in human skin keratinocytes HaCat [10], and did not cause any increase in 8-OHdG level in the genomic DNA and in the expression of DNA damage-associated proteins in human lung epithelial cells A549 [18]. Our previous study also showed that exposure to Nano-TiO_2_ only caused a transient mild inflammation that resolved in a few days post-instillation and did not cause fibrotic changes in mouse lungs [49]. However, conflicting results exist in the available literature regarding the cytotoxic and genotoxic effects of TiO_2_ nanoparticles and the rationale for these is not clear because different cell types, doses, exposure methods, nanoparticle sizes, degree of nanoparticle aggregation, etc. have been used. Moreover, TiO_2_ nanoparticles naturally can occur in three different crystalline forms namely anatase, rutile, and brookite. The Nano-TiO_2_ used in our studies is composed of anatase (90%) and rutile (10%). Previous studies demonstrated that anatase TiO_2_ nanoparticles did not induce any significant neoplastic or genotoxic effects, while rutile TiO_2_ nanoparticles appeared to be slightly genotoxic [86], indicating that different compositions of TiO_2_ nanoparticles may have different toxicities.

## Conclusions

Taken together, this study unraveled the mechanisms underlying Nano-Ni-induced cell malignant transformation. The combined effects of Nano-Ni-induced DNA damage and DNA repair defects through HIF-1α/miR-210/Rad52 pathway probably contribute to Nano-Ni-induced genomic instability and ultimately cell transformation (Fig. 9). However, certain DNA mutations, such as base substitution and simple and small base deletions/insertions, are not the main types of Nano-Ni-induced DNA damage. Nano-Ni-induced DNA DSBs and defects in DNA homologous recombination repair may result in large number of base deletions and complex type of DNA rearrangements by nonhomologous end-joining during repair of DSBs in DNA, which needs to be further studied. Other types of DNA damage, such as DNA modification, DNA adduct formation, etc. also cannot be completely excluded in the process of Nano-Ni-induced cell transformation and need to be further studied. Our findings will provide information to further elucidate the molecular mechanisms of Nano-Ni-induced genotoxicity and carcinogenicity.

## Supplementary Information

**Additional file 1**. Inhibition of or knocking-out HIF-1α did not affect Nano-Ni-induced up-regulation of DNA damage response-associated proteins. (A) BEAS-2B cells were pretreated with 1 µM of 17-AAG for 4 h, followed by treatment with 20 µg/mL of Nano-Ni for 24 h. (B) HIF-1α wild-type (+/+) and knock-out (-/-) cells were treated with 20 µg/mL of Nano-Ni for 24 h. Cells without any treatments were used as control. Nuclear protein was subjected to Western blot. Equal nuclear protein loading was verified by Coomassie Brilliant Blue staining.

**Additional file 2**. Expression of Rad52 in colonies. BEAS-2B cells were transduced with lentiviral particles containing human Rad52 ORF as described in the Methods. 11 puromycin-resistant colonies were picked and expanded for Western blot. The exposure time was 2 sec in panel A and 1 min in panel B. β-actin served as loading control. WT, wild-type.

**Additional file 3**. gpt mutant frequency in mouse lungs. gpt delta transgenic mice were instilled intratracheally with either 50 µg per mouse of Nano-Ni or physiological saline (control). Lung tissues were collected at four months after Nano-Ni instillation. Data are shown as mean ± SEM of 4-5 mice.

## Data Availability

All data and materials are included in the manuscript.

## Abbreviations

Nano-Ni: Nickel nanoparticles
Nano-TiO_2_: Titanium dioxide nanoparticles
DDR: DNA damage response
HIF-1α: Hypoxia-inducible factor 1-alpha
miR-210: MicroRNA-210
HRR: Homologous recombinational repair
DSBs: DNA double strand breaks
ATM: Ataxia telangiectasia, mutated
H2AX: H2A histone family member X
6-TG: 6-Thioguanine
PCNA: Proliferating cell nuclear antigen
17-AAG: 17-(Allylamino)-17-demethoxygeldanamycin

## References

[1] Imran Din M, Rani A (2016). Recent advances in the synthesis and stabilization of nickel and nickel oxide nanoparticles: a green adeptness. Int J Anal Chem..

[2] Mo Y, Jiang M, Zhang Y, Wan R, Li J, Zhong CJ (2019). Comparative mouse lung injury by nickel nanoparticles with differential surface modification. J Nanobiotechnology..

[3] Mo Y, Zhang Y, Wan R, Jiang M, Xu Y, Zhang Q (2020). miR-21 mediates nickel nanoparticle-induced pulmonary injury and fibrosis. Nanotoxicology.

[4] Zhang Q, Kusaka Y, Sato K, Nakakuki K, Kohyama N, Donaldson K (1998). Differences in the extent of inflammation caused by intratracheal exposure to three ultrafine metals: role of free radicals. J Toxicol Environ Health A.

[5] Zhang Q, Kusaka Y, Zhu X, Sato K, Mo Y, Kluz T (2003). Comparative toxicity of standard nickel and ultrafine nickel in lung after intratracheal instillation. J Occup Health.

[6] Dick CA, Brown DM, Donaldson K, Stone V (2003). The role of free radicals in the toxic and inflammatory effects of four different ultrafine particle types. Inhal Toxicol.

[7] Zhang Q, Kusaka Y, Sato K, Mo Y, Fukuda M, Donaldson K (1998). Toxicity of ultrafine nickel particles in lungs after intratracheal instillation. J Occup Health.

[8] Mo Y, Zhang Y, Mo L, Wan R, Jiang M, Zhang Q (2020). The role of miR-21 in nickel nanoparticle-induced MMP-2 and MMP-9 production in mouse primary monocytes: In vitro and in vivo studies. Environ Pollut.

[9] Wan R, Mo Y, Chien S, Li Y, Li Y, Tollerud DJ (2011). The role of hypoxia inducible factor-1alpha in the increased MMP-2 and MMP-9 production by human monocytes exposed to nickel nanoparticles. Nanotoxicology.

[10] Yuan J, Zhang Y, Zhang Y, Mo Y, Zhang Q (2021). Effects of metal nanoparticles on tight junction-associated proteins via HIF-1alpha/miR-29b/MMPs pathway in human epidermal keratinocytes. Part Fibre Toxicol.

[11] Journeay WS, Goldman RH (2014). Occupational handling of nickel nanoparticles: a case report. Am J Ind Med.

[12] Phillips JI, Green FY, Davies JC, Murray J (2010). Pulmonary and systemic toxicity following exposure to nickel nanoparticles. Am J Ind Med.

[13] Kasprzak KS, Sunderman FW, Salnikow K (2003). Nickel carcinogenesis. Mutat Res.

[14] Particulate Matter (PM) Basics. 2021. https://www.epa.gov/pm-pollution/particulate-matter-pm-basics#effects. Accessed 09 Sep 2021.

[15] Pilie PG, Tang C, Mills GB, Yap TA (2019). State-of-the-art strategies for targeting the DNA damage response in cancer. Nat Rev Clin Oncol.

[16] Lord CJ, Ashworth A (2012). The DNA damage response and cancer therapy. Nature.

[17] Shiloh Y, Ziv Y (2013). The ATM protein kinase: regulating the cellular response to genotoxic stress, and more. Nat Rev Mol Cell Biol.

[18] Wan R, Mo Y, Feng L, Chien S, Tollerud DJ, Zhang Q (2012). DNA damage caused by metal nanoparticles: involvement of oxidative stress and activation of ATM. Chem Res Toxicol.

[19] Khanna KK, Keating KE, Kozlov S, Scott S, Gatei M, Hobson K (1998). ATM associates with and phosphorylates p53: mapping the region of interaction. Nat Genet.

[20] Huen MS, Chen J (2008). The DNA damage response pathways: at the crossroad of protein modifications. Cell Res.

[21] Jackson SP, Bartek J (2009). The DNA-damage response in human biology and disease. Nature.

[22] Ivan M, Kondo K, Yang H, Kim W, Valiando J, Ohh M (2001). HIFalpha targeted for VHL-mediated destruction by proline hydroxylation: implications for O2 sensing. Science.

[23] Jaakkola P, Mole DR, Tian YM, Wilson MI, Gielbert J, Gaskell SJ (2001). Targeting of HIF-alpha to the von Hippel-Lindau ubiquitylation complex by O2-regulated prolyl hydroxylation. Science.

[24] Wang GL, Jiang BH, Rue EA, Semenza GL (1995). Hypoxia-inducible factor 1 is a basic-helix-loop-helix-PAS heterodimer regulated by cellular O2 tension. Proc Natl Acad Sci U S A.

[25] Bristow RG, Hill RP (2008). Hypoxia and metabolism. Hypoxia, DNA repair and genetic instability. Nat Rev Cancer.

[26] Maxwell P, Salnikow K (2004). HIF-1: an oxygen and metal responsive transcription factor. Cancer Biol Ther.

[27] Ajdukovic J (2016). HIF-1–a big chapter in the cancer tale. Exp Oncol.

[28] Lewis BP, Burge CB, Bartel DP (2005). Conserved seed pairing, often flanked by adenosines, indicates that thousands of human genes are microRNA targets. Cell.

[29] Latronico MV, Condorelli G (2009). MicroRNAs and cardiac pathology. Nat Rev Cardiol.

[30] Port JD, Sucharov C (2010). Role of microRNAs in cardiovascular disease: therapeutic challenges and potentials. J Cardiovasc Pharmacol.

[31] Ha TY (2011). MicroRNAs in human diseases: from lung, liver and kidney diseases to infectious disease, sickle cell disease and endometrium disease. Immune Netw..

[32] Ha TY (2011). MicroRNAs in human diseases: from cancer to cardiovascular disease. Immune Netw..

[33] Brown D, Rahman M, Nana-Sinkam SP (2014). MicroRNAs in respiratory disease. A clinician's overview. Ann Am Thorac Soc.

[34] Sessa R, Hata A (2013). Role of microRNAs in lung development and pulmonary diseases. Pulm Circ..

[35] Chen S, Xue Y, Wu X, Le C, Bhutkar A, Bell EL (2014). Global microRNA depletion suppresses tumor angiogenesis. Genes Dev.

[36] Gee HE, Ivan C, Calin GA, Ivan M (2014). HypoxamiRs and cancer: from biology to targeted therapy. Antioxid Redox Signal.

[37] Huang X, Ding L, Bennewith KL, Tong RT, Welford SM, Ang KK (2009). Hypoxia-inducible mir-210 regulates normoxic gene expression involved in tumor initiation. Mol Cell.

[38] Camps C, Buffa FM, Colella S, Moore J, Sotiriou C, Sheldon H (2008). hsa-miR-210 Is induced by hypoxia and is an independent prognostic factor in breast cancer. Clin Cancer Res.

[39] McCormick RI, Blick C, Ragoussis J, Schoedel J, Mole DR, Young AC (2013). miR-210 is a target of hypoxia-inducible factors 1 and 2 in renal cancer, regulates ISCU and correlates with good prognosis. Br J Cancer.

[40] Crosby ME, Kulshreshtha R, Ivan M, Glazer PM (2009). MicroRNA regulation of DNA repair gene expression in hypoxic stress. Cancer Res.

[41] Scully R, Panday A, Elango R, Willis NA (2019). DNA double-strand break repair-pathway choice in somatic mammalian cells. Nat Rev Mol Cell Biol.

[42] Sugiyama T, New JH, Kowalczykowski SC (1998). DNA annealing by RAD52 protein is stimulated by specific interaction with the complex of replication protein A and single-stranded DNA. Proc Natl Acad Sci U S A.

[43] Feng Z, Scott SP, Bussen W, Sharma GG, Guo G, Pandita TK (2011). Rad52 inactivation is synthetically lethal with BRCA2 deficiency. Proc Natl Acad Sci U S A.

[44] Lok BH, Carley AC, Tchang B, Powell SN (2013). RAD52 inactivation is synthetically lethal with deficiencies in BRCA1 and PALB2 in addition to BRCA2 through RAD51-mediated homologous recombination. Oncogene.

[45] Sullivan-Reed K, Bolton-Gillespie E, Dasgupta Y, Langer S, Siciliano M, Nieborowska-Skorska M (2018). Simultaneous targeting of PARP1 and RAD52 triggers dual synthetic lethality in BRCA-deficient tumor cells. Cell Rep.

[46] Park MS (1995). Expression of human RAD52 confers resistance to ionizing radiation in mammalian cells. J Biol Chem.

[47] Veerappan I, Sankareswaran SK, Palanisamy R (2019). Morin protects human respiratory cells from PM2.5 induced genotoxicity by mitigating ROS and reverting altered miRNA expression. Int J Environ Res Public Health.

[48] Tessitore A, Cicciarelli G, Del Vecchio F, Gaggiano A, Verzella D, Fischietti M (2014). MicroRNAs in the DNA damage/repair network and cancer. Int J Genomics..

[49] Wan R, Mo Y, Zhang Z, Jiang M, Tang S, Zhang Q (2017). Cobalt nanoparticles induce lung injury, DNA damage and mutations in mice. Part Fibre Toxicol.

[50] Mo Y, Zhu X, Hu X, Tollerud DJ, Zhang Q (2008). Cytokine and NO release from peripheral blood neutrophils after exposure to metal nanoparticles: in vitro and ex vivo studies. Nanotoxicology.

[51] Isaacs JS, Jung YJ, Mimnaugh EG, Martinez A, Cuttitta F, Neckers LM (2002). Hsp90 regulates a von Hippel Lindau-independent hypoxia-inducible factor-1 alpha-degradative pathway. J Biol Chem.

[52] Mo Y, Wan R, Chien S, Tollerud DJ, Zhang Q (2009). Activation of endothelial cells after exposure to ambient ultrafine particles: the role of NADPH oxidase. Toxicol Appl Pharmacol.

[53] Feng L, Zhang Y, Jiang M, Mo Y, Wan R, Jia Z (2015). Up-regulation of Gadd45alpha after exposure to metal nanoparticles: the role of hypoxia inducible factor 1alpha. Environ Toxicol.

[54] Zhang Y, Mo Y, Gu A, Wan R, Zhang Q, Tollerud DJ (2016). Effects of urban particulate matter with high glucose on human monocytes U937. J Appl Toxicol.

[55] Livak KJ, Schmittgen TD (2001). Analysis of relative gene expression data using real-time quantitative PCR and the 2(-Delta Delta C(T)) Method. Methods.

[56] Zhang Q, Salnikow K, Kluz T, Chen LC, Su WC, Costa M (2003). Inhibition and reversal of nickel-induced transformation by the histone deacetylase inhibitor trichostatin A. Toxicol Appl Pharmacol.

[57] Nohmi T, Katoh M, Suzuki H, Matsui M, Yamada M, Watanabe M, et al. A new transgenic mouse mutagenesis test system using Spi- and 6-thioguanine selections. Environ Mol Mutagen. 1996; 28(4):465–70. 10.1002/(SICI)1098-2280(1996)28:4<465::AID-EM24>3.0.CO;2-C. 10.1002/(SICI)1098-2280(1996)28:4<465::AID-EM24>3.0.CO;2-C 8991079

[58] Barlow JH, Rothstein R (2010). Timing is everything: cell cycle control of Rad52. Cell Div.

[59] Benson FE, Baumann P, West SC (1998). Synergistic actions of Rad51 and Rad52 in recombination and DNA repair. Nature.

[60] Mah LJ, El-Osta A, Karagiannis TC (2010). gammaH2AX: a sensitive molecular marker of DNA damage and repair. Leukemia.

[61] Sedelnikova OA, Pilch DR, Redon C, Bonner WM (2003). Histone H2AX in DNA damage and repair. Cancer Biol Ther.

[62] Akerlund E, Cappellini F, Di Bucchianico S, Islam S, Skoglund S, Derr R (2018). Genotoxic and mutagenic properties of Ni and NiO nanoparticles investigated by comet assay, gamma-H2AX staining, Hprt mutation assay and ToxTracker reporter cell lines. Environ Mol Mutagen.

[63] Alarifi S, Ali D, Alakhtani S, Al Suhaibani ES, Al-Qahtani AA (2014). Reactive oxygen species-mediated DNA damage and apoptosis in human skin epidermal cells after exposure to nickel nanoparticles. Biol Trace Elem Res.

[64] Magaye R, Gu Y, Wang Y, Su H, Zhou Q, Mao G (2016). In vitro and in vivo evaluation of the toxicities induced by metallic nickel nano and fine particles. J Mol Histol.

[65] Ahamed M, Alhadlaq HA (2014). Nickel nanoparticle-induced dose-dependent cyto-genotoxicity in human breast carcinoma MCF-7 cells. Onco Targets Ther.

[66] Latvala S, Vare D, Karlsson HL, Elihn K (2017). In vitro genotoxicity of airborne Ni-NP in air-liquid interface. J Appl Toxicol.

[67] Latvala S, Hedberg J, Di Bucchianico S, Moller L, Odnevall Wallinder I, Elihn K (2016). Nickel release, ROS generation and toxicity of Ni and NiO Micro- and nanoparticles. PLoS ONE.

[68] Mo Y, Zhang Y, Zhang Q (2019). Evaluation of pulmonary toxicity of nanoparticles by bronchoalveolar lavage. Methods Mol Biol.

[69] Pietruska JR, Liu X, Smith A, McNeil K, Weston P, Zhitkovich A (2011). Bioavailability, intracellular mobilization of nickel, and HIF-1alpha activation in human lung epithelial cells exposed to metallic nickel and nickel oxide nanoparticles. Toxicol Sci.

[70] Salnikow K, Donald SP, Bruick RK, Zhitkovich A, Phang JM, Kasprzak KS (2004). Depletion of intracellular ascorbate by the carcinogenic metals nickel and cobalt results in the induction of hypoxic stress. J Biol Chem.

[71] Karaczyn A, Ivanov S, Reynolds M, Zhitkovich A, Kasprzak KS, Salnikow K (2006). Ascorbate depletion mediates up-regulation of hypoxia-associated proteins by cell density and nickel. J Cell Biochem.

[72] Costa M, Davidson TL, Chen H, Ke Q, Zhang P, Yan Y (2005). Nickel carcinogenesis: epigenetics and hypoxia signaling. Mutat Res.

[73] Takahashi S, Takeda E, Kubota Y, Okayasu R (2000). Inhibition of repair of radiation-induced DNA double-strand breaks by nickel and arsenite. Radiat Res.

[74] Arita A, Munoz A, Chervona Y, Niu J, Qu Q, Zhao N (2013). Gene expression profiles in peripheral blood mononuclear cells of Chinese nickel refinery workers with high exposures to nickel and control subjects. Cancer Epidemiol Biomarkers Prev.

[75] Scanlon SE, Scanlon CD, Hegan DC, Sulkowski PL, Glazer PM (2017). Nickel induces transcriptional down-regulation of DNA repair pathways in tumorigenic and non-tumorigenic lung cells. Carcinogenesis.

[76] Magaye R, Zhou Q, Bowman L, Zou B, Mao G, Xu J (2014). Metallic nickel nanoparticles may exhibit higher carcinogenic potential than fine particles in JB6 cells. PLoS ONE.

[77] Gliga AR, Di Bucchianico S, Akerlund E, Karlsson HL (2020). Transcriptome profiling and toxicity following long-term, low dose exposure of human lung cells to Ni and NiO nanoparticles-comparison with NiCl2. Nanomaterials (Basel)..

[78] Scholzen T, Gerdes J. The Ki-67 protein: from the known and the unknown. J Cell Physiol. 2000;182(3):311–22. 10.1002/(SICI)1097-4652(200003)182:3<311::AID-JCP1>3.0.CO;2-9. 10.1002/(SICI)1097-4652(200003)182:3<311::AID-JCP1>3.0.CO;2-9 10653597

[79] Cuylen S, Blaukopf C, Politi AZ, Muller-Reichert T, Neumann B, Poser I (2016). Ki-67 acts as a biological surfactant to disperse mitotic chromosomes. Nature.

[80] Essers J, Theil AF, Baldeyron C, van Cappellen WA, Houtsmuller AB, Kanaar R (2005). Nuclear dynamics of PCNA in DNA replication and repair. Mol Cell Biol.

[81] Boehm EM, Gildenberg MS, Washington MT (2016). The many roles of PCNA in eukaryotic DNA replication. Enzymes..

[82] Bartek J (2011). DNA damage response, genetic instability and cancer: from mechanistic insights to personalized treatment. Mol Oncol.

[83] Levine MS, Holland AJ (2018). The impact of mitotic errors on cell proliferation and tumorigenesis. Genes Dev.

[84] Clift MJD, Doak SH (2021). Advanced in vitro models for replacement of animal experiments. Small.

[85] Halappanavar S, Nymark P, Krug HF, Clift MJD, Rothen-Rutishauser B, Vogel U (2021). Non-animal strategies for toxicity assessment of nanoscale materials: role of adverse outcome pathways in the selection of endpoints. Small.

[86] Uboldi C, Urban P, Gilliland D, Bajak E, Valsami-Jones E, Ponti J (2016). Role of the crystalline form of titanium dioxide nanoparticles: rutile, and not anatase, induces toxic effects in Balb/3T3 mouse fibroblasts. Toxicol In Vitro.

