# Supplementary figures and images for: Nickel nanoparticle-induced cell transformation: involvement of DNA damage and DNA repair defect through HIF-1α/miR-210/Rad52 pathway
Source: J Nanobiotechnology. 2021 Nov 17;19:370. doi: 10.1186/s12951-021-01117-7 (PMC8600818; doi:10.1186/s12951-021-01117-7)

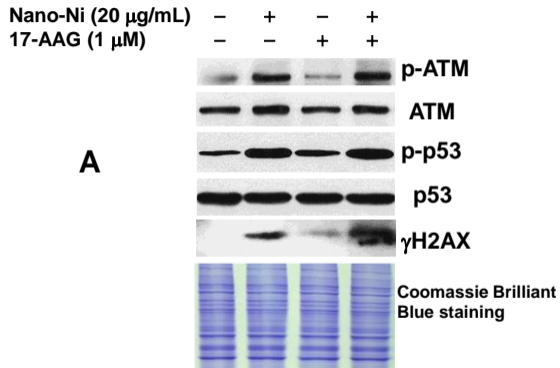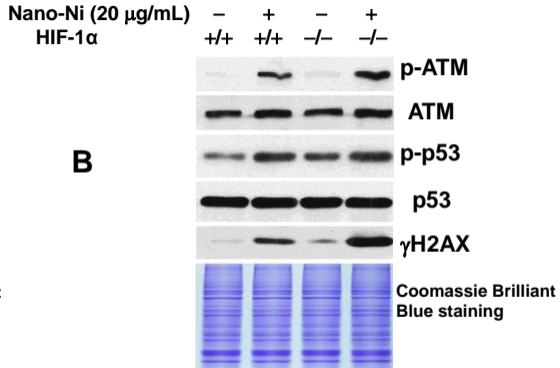

Supplement: Supplementary file 1 — Additional file 1. Inhibition of or knocking-out HIF-1α did not affect Nano-Ni-induced up-regulation of DNA damage response-associated proteins. (A) BEAS-2B cells were pretreated with 1 µM of 17-AAG for 4 h, followed by treatment with 20 µg/mL of Nano-Ni for 24 h. (B) HIF-1α wild-type (+/+) and knock-out (-/-) cells were treated with 20 µg/mL of Nano-Ni for 24 h. Cells without any treatments were used as control. Nuclear protein was subjected to Western blot. Equal nuclear protein loading was verified by Coomassie Brilliant Blue staining. [file 12951_2021_1117_MOESM1_ESM.pdf]

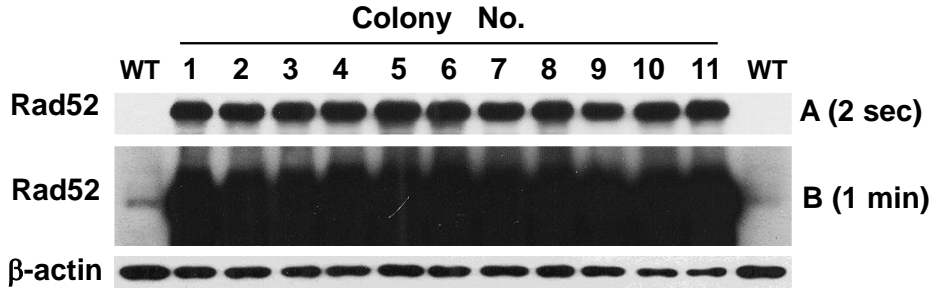

Supplement: Supplementary file 2 — Additional file 2. Expression of Rad52 in colonies. BEAS-2B cells were transduced with lentiviral particles containing human Rad52 ORF as described in the Methods. 11 puromycin-resistant colonies were picked and expanded for Western blot. The exposure time was 2 sec in panel A and 1 min in panel B. β-actin served as loading control. WT, wild-type. [file 12951_2021_1117_MOESM2_ESM.pdf]

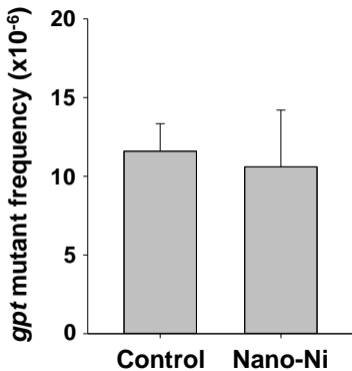

Supplement: Supplementary file 3 — Additional file 3. gpt mutant frequency in mouse lungs. gpt delta transgenic mice were instilled intratracheally with either 50 µg per mouse of Nano-Ni or physiological saline (control). Lung tissues were collected at four months after Nano-Ni instillation. Data are shown as mean ± SEM of 4-5 mice. [file 12951_2021_1117_MOESM3_ESM.pdf]
